# Quality assurance for online adaptive radiotherapy: a secondary dose verification model with geometry-encoded U-Net

**DOI:** 10.1088/2632-2153/ad829e

**Published:** 2024-10-11

**Authors:** Shunyu Yan, Austen Maniscalco, Biling Wang, Dan Nguyen, Steve Jiang, Chenyang Shen

**Affiliations:** The Medical Artificial Intelligence and Automation (MAIA) Laboratory, Department of Radiation Oncology, University of Texas Southwestern Medical Center, Dallas, TX 75390, United States of America

**Keywords:** secondary dose verification, real-time calculation, artificial intelligence, deep learning, prostate cancer

## Abstract

In online adaptive radiotherapy (ART), quick computation-based secondary dose verification is crucial for ensuring the quality of ART plans while the patient is positioned on the treatment couch. However, traditional dose verification algorithms are generally time-consuming, reducing the efficiency of ART workflow. This study aims to develop an ultra-fast deep-learning (DL) based secondary dose verification algorithm to accurately estimate dose distributions using computed tomography (CT) and fluence maps (FMs). We integrated FMs into the CT image domain by explicitly resolving the geometry of treatment delivery. For each gantry angle, an FM was constructed based on the optimized multi-leaf collimator apertures and corresponding monitoring units. To effectively encode treatment beam configuration, the constructed FMs were back-projected to $30$ cm away from the isocenter with respect to the exact geometry of the treatment machines. Then, a 3D U-Net was utilized to take the integrated CT and FM volume as input to estimate dose. Training and validation were performed on $381$ prostate cancer cases, with an additional $40$ testing cases for independent evaluation of model performance. The proposed model can estimate dose in ∼$15$ ms for each patient. The average *γ* passing rate ($3\% /2\,{\text{mm}}$, $10\% $ threshold) for the estimated dose was 99.9% ± 0.15% on testing patients. The mean dose differences for the planning target volume and organs at risk were $0.07\% \pm 0.34\% $ and $0.48\% \pm 0.72\% $, respectively. We have developed a geometry-resolved DL framework for accurate dose estimation and demonstrated its potential in real-time online ART doses verification.

## 1. Introduction

Conventional radiotherapy (RT) employs a fixed treatment plan that was carefully developed based on a computed tomography (CT) image of the patient acquired at RT simulation. Due to day-to-day patient anatomical changes and setup uncertainties, maintaining the accuracy and reproducibility of patient positioning in subsequent daily RT treatment can be a challenging task [1]. Geometrical changes to the patient’s daily anatomy—due to things such as variability in patient setup, tumor shrinkage, patient weight change, gastrointestinal movement, etc—leads to deteriorated target coverage and organs-at-risk (OARs) sparing [2]. To ensure adequate tumor control while minimizing the risks of normal tissue complications, online adaptive RT (ART) has recently been introduced into routine clinical practice to adjust RT treatment plans according to patient positioning and anatomical changes immediately before each RT treatment [3].

RT plan quality assurance (QA) is essential to verify the dose of online ART plan is essential to the safety and effectiveness of the treatment plan’s dose delivery [4]. The most commonly used dose verification approach for conventional RT plan QA is to perform physical dose measurement and compare the result with the planned dose [5–7]. However, measurement-based QA might not be feasible for online ART as it would disrupt the adaptive workflow by having to remove the patients from the couch for QA delivery and thereby introducing additional anatomical and positioning uncertainties [8]. To address this issue, computation-based dose verification can be employed to replace measurement-based approach. Conventional computation-based dose verification algorithms such as analytical anisotropic algorithm [9], collapsed cone convolution algorithm [10], and Monte-Carlo (MC) algorithm [11–13] often require extensive computational resources and time [14–19]. For instance, in Piffer *et al* [17], a central processing unit-based MC secondary dose check tool takes in average $30 \pm 12$ min of calculation per treatment plan, which extensively prolongs the time patient staying on the treatment couch in online ART. In Beltran *et al* [16], their clinical implementation of graphic processing unit (GPU)-based MC required about $15$ min per treatment plan verification, posing challenges for time-sensitive online ART. The long waiting time can further result in substantial changes in patient positioning and anatomy as well as patient discomfort [20]. Accelerated computation-based dose verification algorithm is strongly desired to mitigate these uncertainties.

Artificial intelligence, particularly deep learning (DL), has been making transformative strides in RT, offering automation and acceleration of complex procedures with an efficiency that is typically several orders of magnitude higher [21]. Recent studies have been conducted to utilize the power of DL for the purpose of expediting dose calculation for both photon and proton particles. For proton therapy, Nomura *et al* developed a rapid spot scanning proton dose calculation method by converting spot beam data and single-filed uniform dose labels into a fixed-size ‘peak map’ as prior information for dose prediction using a 3D convolutional neural network [22]. Meanwhile, Wang *et al* introduced a recurrent U-Net based method for intensity-modulated proton therapy dose prediction [23]. In realm of DL-based dose calculation for photon therapy, Xing *et al* utilized broad beam ray-tracing algorithm to project fluence maps (FMs) to dose domain as an initial dose calculation input for a U-Net to predict final dose distribution [24]. Another work done by Wu *et al* employed the pencil beam (PB) dose and CT images as input to a network for the purpose of generating the MC dose, which boosted the accuracy of PB dose while maintaining its fast speed [25]. Despite their great success in both proton and photon therapy dose estimation, these algorithms still require some rough initial dose calculation, for instance, prior spot beam dose label pre-processing for proton therapy, and ray-tracing or PB algorithms for photon beam as the input to the DL models [22–27], which can take minutes of computational time for complicated treatments such as volumetric modulated arc therapy (VMAT). This dose pre-calculation process reduces the practical value of these DL-based dose verification algorithms for the time-sensitive online ART treatment.

To further speed up the dose verification in online ART, this study introduces a geometry-encoded DL-based model for real-time secondary dose verification for VMAT. Specifically, the proposed approach facilitates a simple and unified representation of CT and FMs in the CT image domain, preserving geometrical information of beam delivery characteristics. This representation of CT and FMs is constructed using lightweight computation, allowing for nearly instantaneous derivation. The integrated volume with beam geometry explicitly encoded serves as the input for a 3D U-Net to carry out accurate dose estimation in real-time, eliminating the requirement of time-consuming pre-processing techniques such as ray-tracing or PB-based dose calculation. For proof-of-principle purposes, we focused on prostate cancer patients treated with the VMAT technique to validate the effectiveness of the proposed algorithm and the dose prediction was performed only to the central axial slice. To our knowledge, this is the first study that directly encodes beam delivery geometry in real-time for online ART dose verification. This approach can also be easily generalized for 3D dose prediction without sacrificing too much efficiency as well as for other disease sites and treatment modalities, offering rapid dose verification for different RT workflows.

## 2. Methods

### 2.1. Proposed workflow

**Figure 1.**
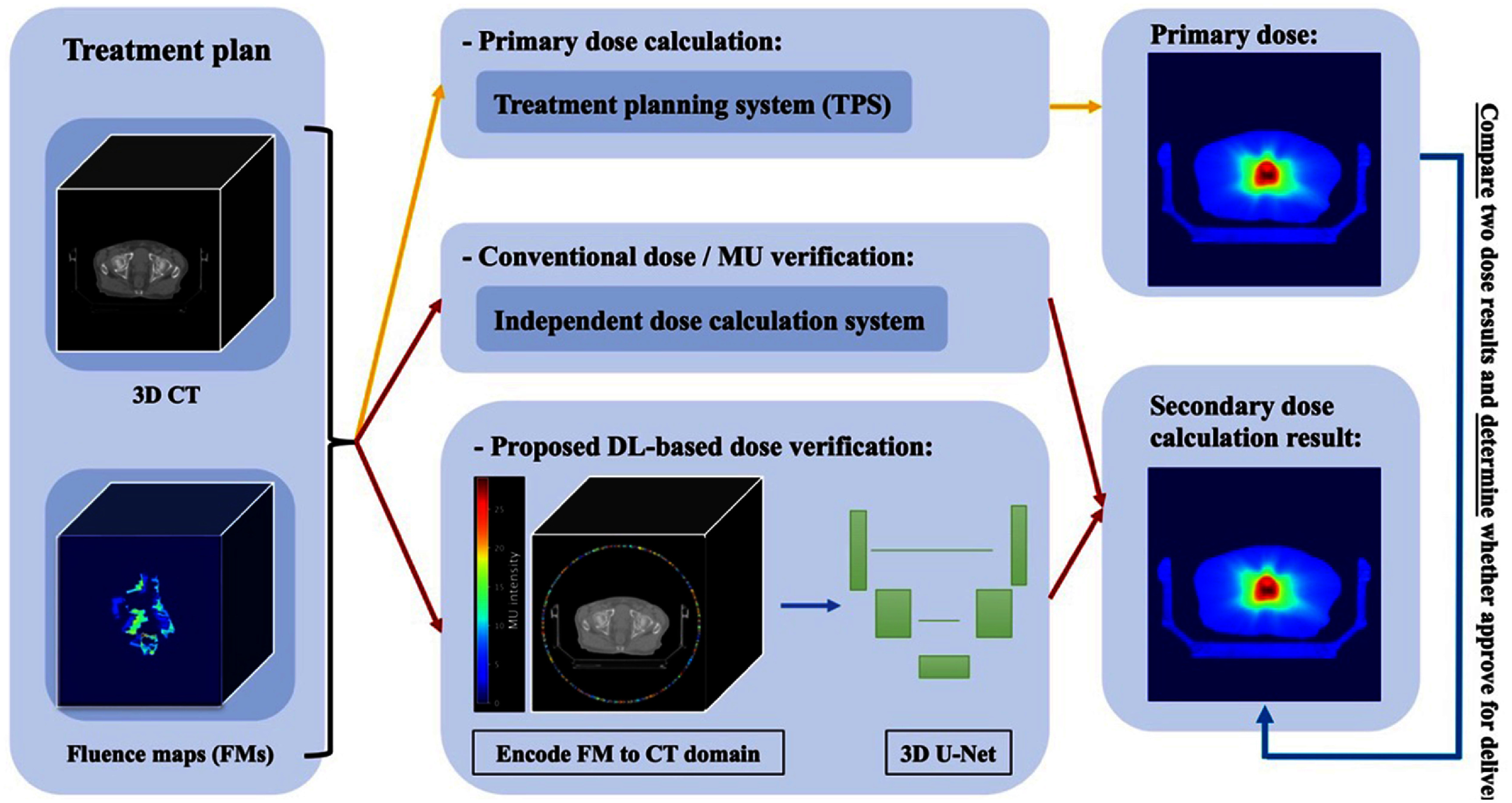
Clinical secondary dose verification workflow according to TG-219 guidelines [8] (upper route) vs proposed DL-based secondary dose verification workflow by using an integrated volume encoding FMs into CT domain as input for 3D U-Net (lower route).

Figure 1 depicts how we can plug in the proposed algorithm seamlessly into the clinical ART workflow in lieu of conventional computational based dose verification. In the clinical workflow of online ART, the process begins with the acquisition of real-time images, typically using cone beam CT, which are used to adapts the treatment plans based on the patient’s anatomy and positioning right before the treatment starts. Following the creation of new treatment plan, the corresponding new FMs are then extracted and reconstructed according to the multi-leaf collimator positions and monitoring unit values. These FMs are then integrated with CT volume via the proposed geometry encoding scheme and then fed directly into our proposed DL model to predict the dose distribution, which can then be employed as the secondary dose calculation result for the purpose of online ART patient-specific QA. In the following sections, we will describe data collection, the geometric encoding process, and the proposed DL framework.

### 2.2. Patient data

In this study, we collected a retrospective cohort of $421$ prostate patients previously treated between 12/2014 and 07/2022 in our clinic with the VMAT technique. To demonstrate the versatility of our model across various cases, we included both stereotactic body radiation therapy (SBRT) and conventional fractioned RT plans. More specifically, among $421$ patient cases, $164$ were treated with SBRT. Among them, $138$ were treated using fraction scheme of $45$ Gy in $5$ fractions while the rest received $40$ Gy in $5$ fractions. For the $257$ conventional plans that were used in this study, the total prescription dose (Rx) ranged from $20$ Gy to $80$ Gy with fractionation schemes from $14$ to $44$. Dose, CT, and FMs were extracted from digital imaging and communications in medicine (DICOM) files and converted into 3D numerical Python arrays. More specifically, 3D Dose distributions were reconstructed from the DICOM files and converted into 3D Python arrays in units of Gy. CT image data was converted from Hounsfield Units (HU) to relative electron density (RED) using the HU-RED calibration curve established in our clinic.

A FM is a representation of the intensity pattern of an x-ray beam modulated across the treatment field of RT. It is always defined in a plane perpendicular to treatment beam, where the treatment isocenter is located. For VMAT, radiation dose is given to patient by rotating the treatment gantry head of a linear accelerator (LINAC) around the patient body with dynamically changing FM carefully optimized to deliver clinically favorable dose distribution. FM at each gantry angle is often expressed in a matrix form where each element corresponds to the intensity of the radiation beam in that specific sub-area of the treatment field [28]. It is reconstructed directly according to the machine parameters recorded in a radiation therapy treatment plan. Note that a VMAT treatment plan may consist of multiple treatment arcs and in our study the FMs from different treatment arcs were summed together for each gantry angle to represent the complete treatment plan.

We randomly selected $40$ out of $421$ total patients to be reserved as the independent testing dataset. From the remaining $381$ patients, approximately $80\% $ ($304$ patient cases) were employed as training cases and the rest ($77$ cases) were used to calculate validation loss during the model training process.

### 2.3. Geometry encoding process

**Figure 2.**
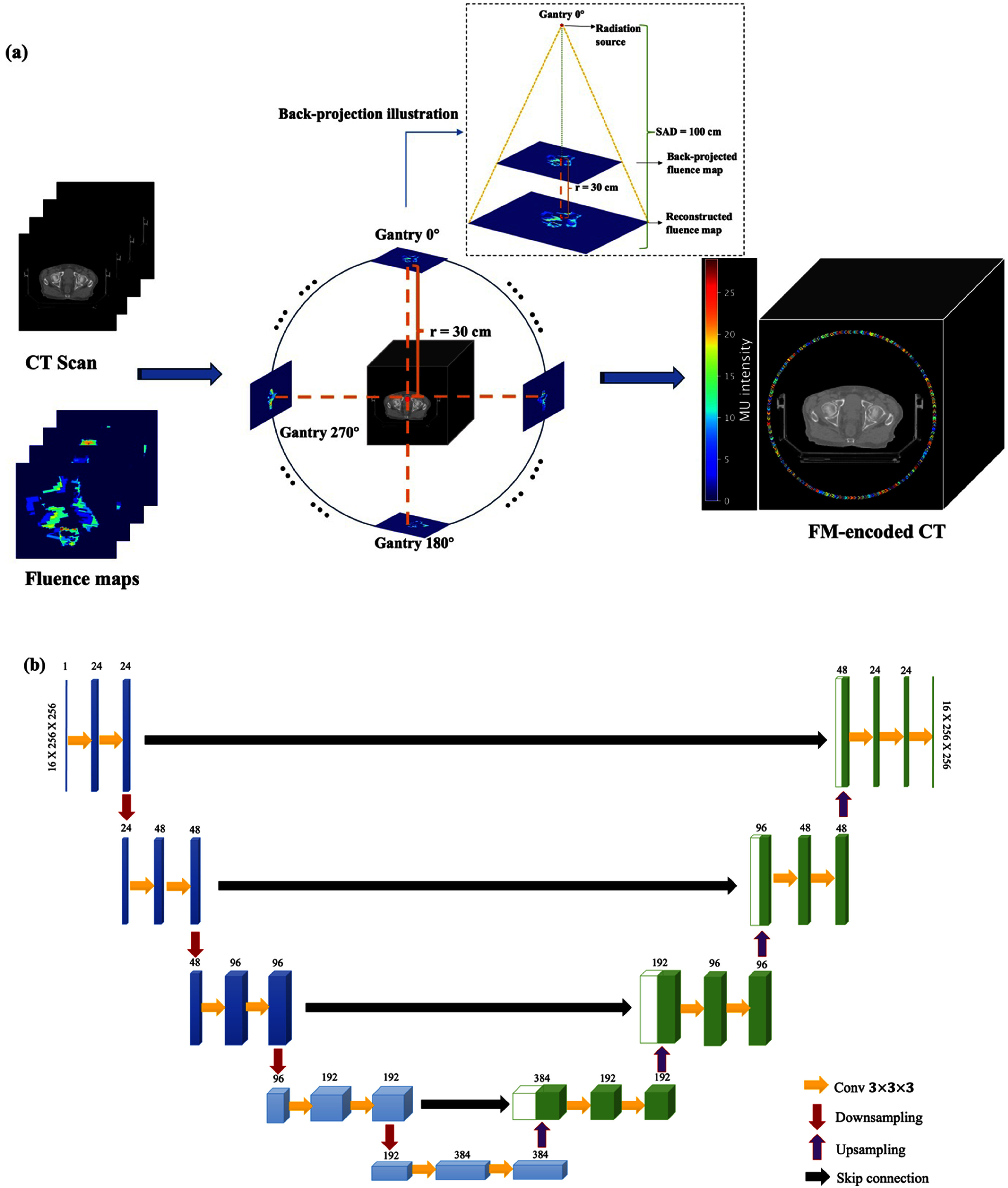
(a) Illustration of encoding FMs to the patient CT image domain. (b) Schematic diagram of proposed 3D U-Net module.

The key innovation in this study is the precise spatial encoding of FMs into the CT imaging domain, which captures the exact beam delivery geometry in ART. More specifically, we consider VMAT delivery where FMs were defined at every two degrees of gantry angle, resulting in total of $180$ FMs for a whole treatment arc. In the CT image domain, FMs were originally defined at treatment isocenter. Then, according to the gantry angle of each control point, an FM was back-projected from isocenter to a fixed distance by assuming a point source projection geometry with $100$ cm source-to-axis distance, matching the geometry of LINACs used for plans in this study. To ensure the FM is outside the patient body and the treatment couch while preserving the delivery geometry, a distance of was $30\,{\text{cm}}$ from treatment isocenter was chosen (see figure 2(a)). This configuration effectively simulates the beam delivery by integrating the external beam treatment setup based on FMs with the anatomical information from CT. Due to the large size of the integrated volumes, we re-sampled all the data to a resolution of ${ }2.7\,{\text{mm}}\, \times \,2.7\,{\text{mm}}\, \times \,3\,{\text{mm}}$, resulting in volumes in a dimension of ${ }256\, \times \,256\, \times \,96$. As a proof-of-principle study, we evaluate the feasibility of dose estimation using only the central axial slice. Specifically, for each patient, a sub-volume centered at treatment isocenter with a dimension of $256 \times 256 \times 16$ was extracted as input. The physical length of *z* dimension for each sub-volume was $48\,{\text{mm}}$ to account for scatter along patient superior–inferior direction for accurate dose estimation. It is worth mentioning that the proposed method can be easily extended for direct 3D dose estimation at a cost of higher video random access memory and computational burden on GPUs during the model training stage.

### 2.4. DL

#### 2.4.1. Network structure

We aim to establish a DL-based model that can predict accurate dose distributions based on FM-encoded CT images for online ART secondary dose verification. Our network architecture employed in this study was inspired by the U-Net due to its promising performance in a wide range of image-to-image prediction tasks for applications in different disciplines [29–32]. The detailed network architecture is illustrated in figure 2(b) [33].

More specifically, four convolutional blocks were implemented for each half of the U-net. For the first half, i.e. the descending arm, each block consists of two convolutional layers followed by a downsampling operation. The kernel size in all convolutional layers is set as $3 \times 3 \times 3$. Inspired by the Concatenated ReLU (CReLU) activation function [34], which preserves positive and negative information by concatenating the feature map channels from a positive and negative ReLU function, the Convolved CReLU activation function uses a convolutional layer after CReLU so that the number of feature map channels can be reduced back down. Group normalization was then employed after the activation layer. For downsampling, our model employed both 3D max-pooling and 3D average-pooling, after which the outputs are concatenated. The U-Net starts with $24$ initial feature maps which are doubled after each downsampling block. The second half, i.e. the ascending arm, was implemented essentially by mirroring the descending arm. In contrast to the two pooling operators used for downsampling in the first half, nearest-neighbor interpolation and a trilinear interpolation are incorporated in the second half of the U-Net for upsampling. Skip connections were also implemented between the blocks on the same level of ascending and descending arms to propagate high resolution features.

#### 2.4.2. Training strategy

The loss function we utilized in this study was mean square error with the AdamW algorithm [35] as the optimizer for model training. We used 3D DropBlock for network regularization with a maximal block size of $5 \times 5 \times 5$ and a dropout rate of $0.1$. Batch size was set to $4$ and the initial learning rate was set as $5 \times {10^{ - 4}}$ in the training process. As we focus on estimating dose for central axial slice in this proof-of-principle study, during model training we only computed the loss for that slice. The model was trained until convergence, which was defined as no decreases in the loss function value within $30$ epochs. The training was conducted on a single NVIDIA V$100$ GPU with $32$ GB of RAM.

#### 2.4.3. Data augmentation

Without any data augmentation, there is a potential risk that the model could completely rely on patient anatomy and electron density values in CT data for dose estimation, ignoring the contribution from the FMs which is critically important in dose verification. To enhance the effectiveness of training and improve the capability of the model in learning the dose deposition principles, we employed several strategies for data augmentation to encourage the importance of FMs. The first strategy we introduced is to scale FM intensity and dose together with a random factor within [0, 5] while preserving the original electron density values in CT images unchanged. This was implemented to emphasize the correlation between FMs and the dose distributions in model training. On top of this strategy to emphasize the effect of FMs on the final dose, we have also incorporated rotation operation at random angles applied to both FM-encoded CT images and corresponding dose distributions to create more training samples to improve training performance.

### 2.5. Analysis

To evaluate the performance of the proposed model, the trained model estimated the dose distributions for independent testing cases based on the input FM-encoded CT images. Then, the difference between estimated and calculated dose was investigated using both dose error maps and dose-volume-histograms (DVHs). To further assess the accuracy of the proposed DL-based dose estimation model quantitatively, we compared the commonly used dosimetric criteria derived from estimated and calculated dose, respectively. The dosimetric evaluation of interest included ${D_{{{98\% }}}}$, ${D_{95\% }}$, ${D_{{\text{max}}}}$, ${D_{{\text{min}}}}$, and ${D_{{\text{mean}}}}$ for planning target volume (PTV), and ${D_{{\text{mean}}}}$ and ${D_{{\text{max}}}}$ for relevant OARs. Moreover, we utilized the open-source PyMedPhys python library [36] to perform $\gamma $ analysis using both $3{{\% }}/2\,{\text{mm}}$ and $2{{\% }}/2\,{\text{mm}}$ criteria with global normalization and a threshold of $10{{\% }}$ of the maximum dose.

## 3. Results

**Figure 3.**
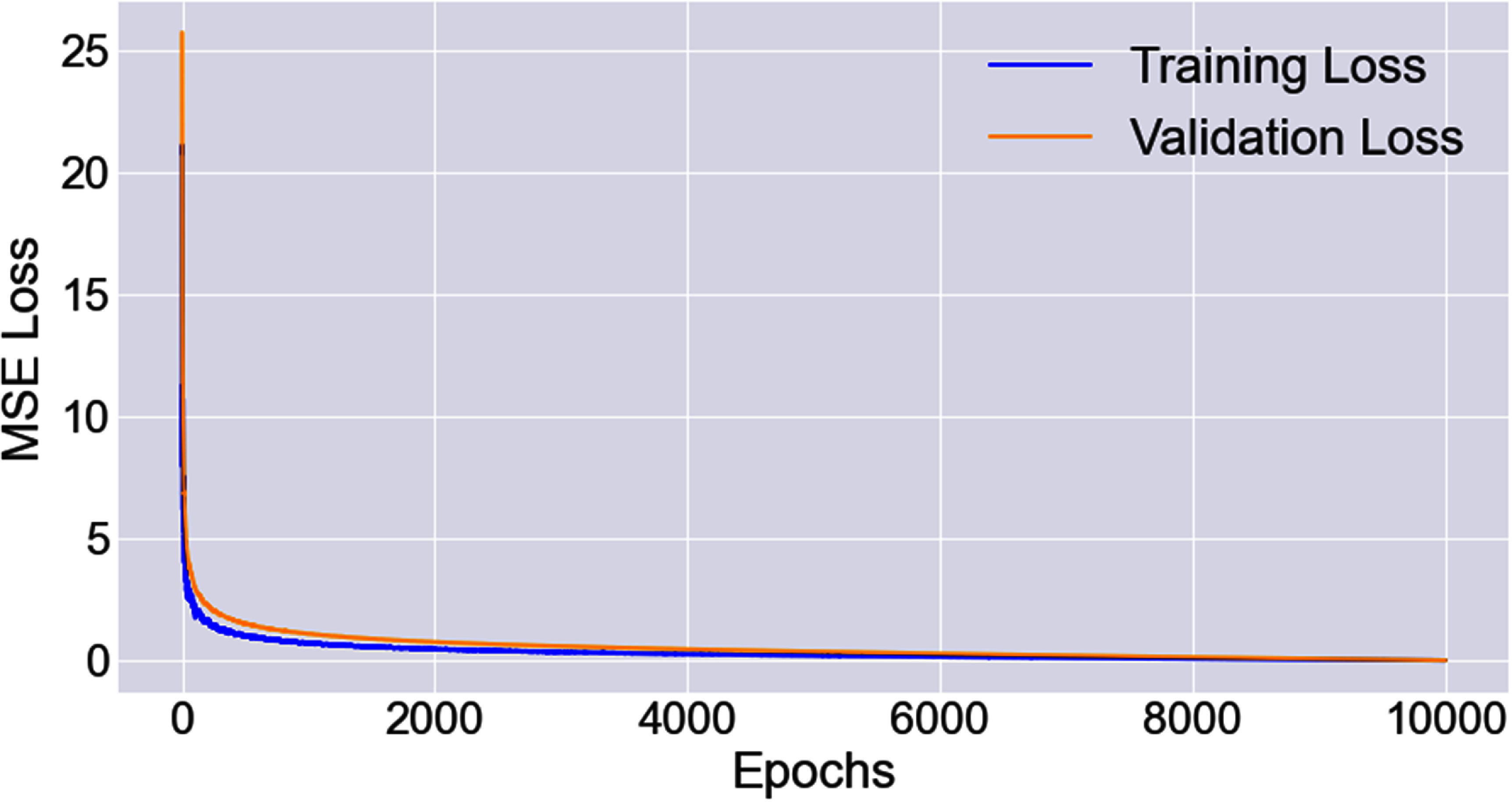
Training and validation loss of the proposed model along training epochs.

The model was trained for $10000{\text{ }}$ epochs, which took approximately $14$ d at a rate of ∼$3$ min per epoch. Generally, both the training loss and validation loss decreased along training epochs and no significant overfitting was observed (see figure 3). The model achieved lowest validation loss was selected for the performance evaluation purpose on testing dataset.

The average time needed to perform secondary dose verification for each patient case using the established model was $14.7$ ms. Given that encoding FMs into the CT image domain, takes an average of $8.4\,{\text{ms}}$ per patient, the overall time expected for dose estimation using the proposed framework for a testing patient case is approximately $23.1\,{\text{ms}}$.

**Figure 4.**
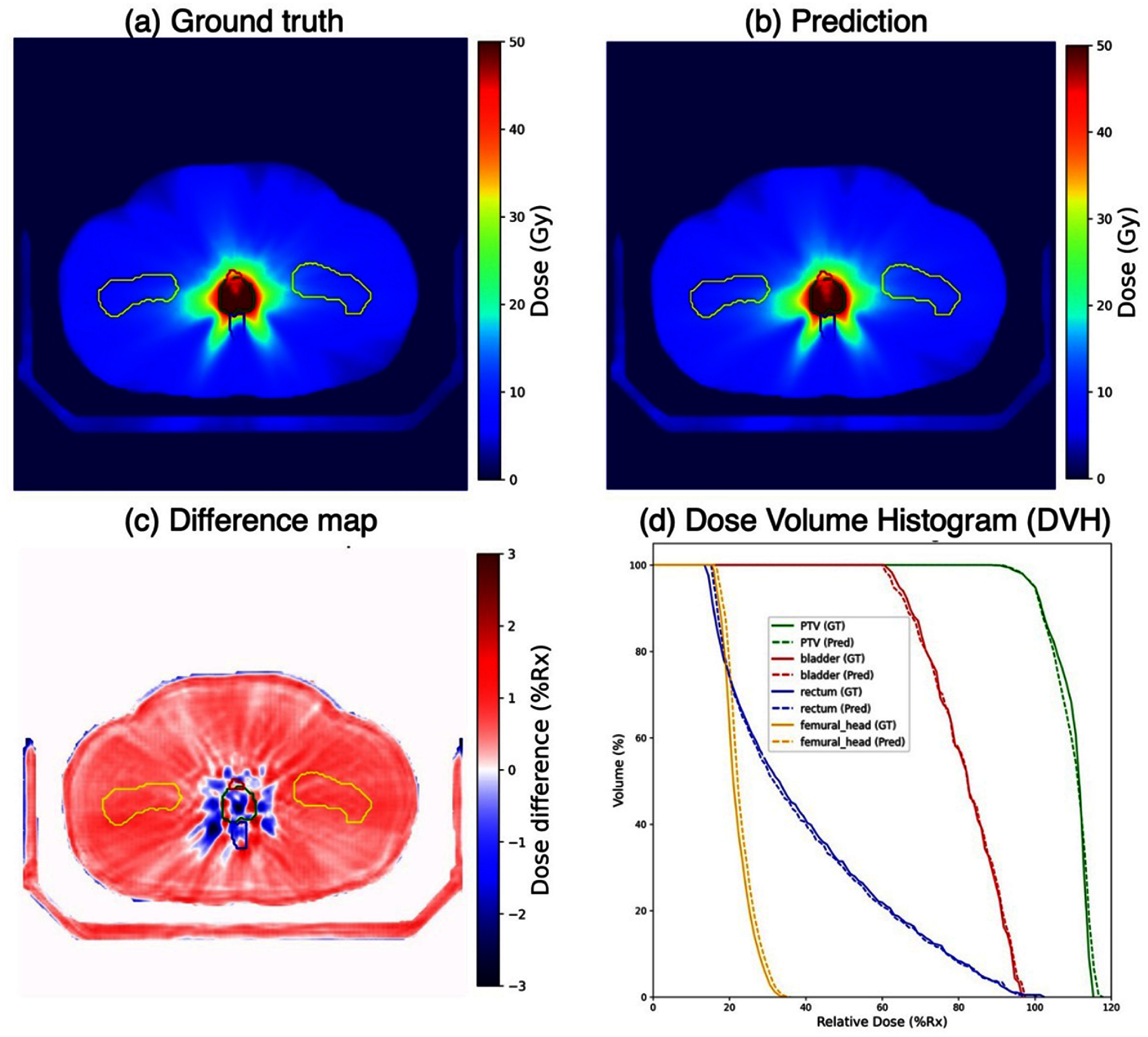
Model performance on a representative testing case. (a)–(c) Ground truth dose distribution, estimated dose distribution, and their difference map (display window $\left[ { - 3{{\% }},{\text{ }}3{{\% }}} \right]$ of Rx. i.e. 45 Gy) with PTV (green), bladder (red), rectum (blue), and femoral heads (yellow) contours; (d) DVH plots of ground truth (solid) and predicted dose (dashed).

Figure 4 depicts the calculated dose distribution (figure 4(a)), estimated dose distribution (figure 4(b)), dose difference map (figure 4(c)), and DVH plots (figure 4(d)) for a representative case from the independent testing set. An excellent agreement between estimated and calculated dose was achieved using the proposed framework particularly for the high dose region, which is of clinical interest.

**Figure 5.**
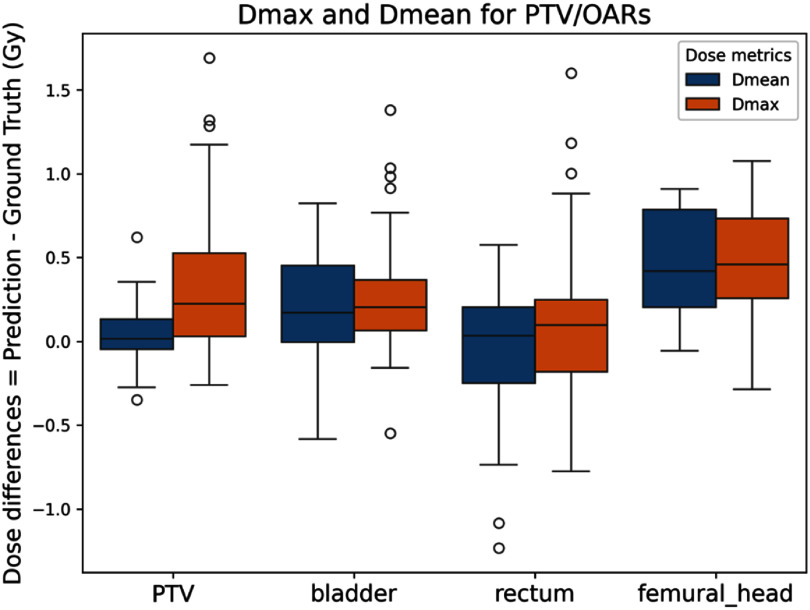
Boxplot comparisons of dose differences for and in PTV, bladder, rectum, and femoral heads.

In addition, quantitative evaluations demonstrated excellent accuracy in dose estimation with the proposed method. Across all testing cases, the average $\gamma $ passing rate using $3{{\% }}/2$ mm and $2\% /2$ mm criteria (10% threshold) was $99.9{{\% }} \pm 0.15{{\% }}$ and $97.32{{\% }} \pm 2.95{{\% }}$ respectively. The detailed quantitative results are summarized in table 1. ${D_{98\% }}$, ${D_{95\% }}$, ${D_{{\text{mean}}}}$, and *D*_min_ for PTV were all within 0$.5\% $ comparing estimated and calculated dose except for ${D_{{\text{max}}}}$. For relevant OARs, evaluation results of ${D_{{\text{mean}}}}$ and ${D_{{\text{max}}}}$ indicate the dose differences were within $0.5$ Gy in average for all OARs and lower than 0.5% error for bladder and rectum, except for femoral heads. Dose differences of ${D_{{\text{max}}}}$ and ${D_{{\text{mean}}}}$ for PTV and associated OARs are visualized in boxplot in figure 5. An overall good alignment of dose distribution was observed in testing patient cases. To test the effectiveness of the proposed algorithm, FMs were removed in all testing data, which means only CT scans were used as model input and near zero dose was expected as output. Although our model did not predict the perfect zero dose results, it managed to generate an average dose of $0.02 \pm 0.17\,{\text{Gy}}$ across all testing cases.

**Table 1.** Dosimetric evaluation of model performance on the testing dataset. For absolute dose error, mean and range of dose error are given. Relative dose error is computed as ${\text{ }}\frac{{{\text{Estrimation}} - {\text{Groundtruth}}}}{{{\text{Prescription Dose }}}} \times 100\% $. Mean and corresponding standard deviation of each dosimetric quantity are provided.

Target	Metrics	Absolute dose error (Gy)	Relative dose error (%)
PTV	${D_{98\% }}$	$0.18{\text{ }}\left( { - 0.15 - 0.69{\text{ }}} \right)$	$0.32 \pm 0.33$
	${D_{95\% }}$	$0.05{\text{ }}\left( { - 0.22 - 0.32{\text{ }}} \right)$	$0.08 \pm 0.21$
	${D_{{\text{max}}}}$	$0.36{\text{ }}\left( { - 0.26 - 1.69{\text{ }}} \right)$	$0.58 \pm 0.76$
	${D_{{\text{min}}}}$	$0.24{\text{ }}\left( { - 0.61 - 0.93{\text{ }}} \right)$	$0.44 \pm 0.66$
	${D_{{\text{mean}}}}$	$0.04{\text{ }}\left( { - 0.35 - 0.62{\text{ }}} \right)$	$0.07 \pm 0.34$
Bladder	${D_{{\text{max}}}}$	$0.26{\text{ }}\left( { - 0.55 - 1.37{\text{ }}} \right)$	$0.46 \pm 0.57$
	${D_{{\text{mean}}}}$	$0.21{\text{ }}\left( { - 0.58 - 0.82{\text{ }}} \right)$	$0.42 \pm 0.60$
Rectum	${D_{{\text{max}}}}$	$0.12{\text{ }}\left( { - 0.77 - 1.61{\text{ }}} \right)$	$0.22 \pm 0.74$
	${D_{{\text{mean}}}}$	$ - 0.05{\text{ }}\left( { - 1.23 - 0.57{\text{ }}} \right)$	$ - 0.02 \pm 0.72$
Femoral Heads	${D_{{\text{max}}}}$	$0.45{\text{ }}\left( { - 0.28 - 1.08{\text{ }}} \right)$	$0.96 \pm 0.81$
	${D_{{\text{mean}}}}$	$0.47{\text{ }}\left( { - 0.05 - 0.91{\text{ }}} \right)$	$1.01 \pm 0.81$

## 4. Discussion

DL has been extensively utilized to forge groundbreaking solutions for many critical tasks in RT, including image quality enhancement [37], target/OARs segmentation [38, 39], and dose prediction [30, 40–43]. Several relevant studies have also been focused on the development of DL algorithm for dose calculation [24–26, 44–46]. Nevertheless, most of the current DL-based dose calculation model still require computationally heavy data preprocessing and extensive post-processing computations such as ray-tracing or PB dose calculation algorithms to generate input data for model training. This large computational demand is challenging for procedures with stringent time constraints, such as online ART.

This study designed a unified representation for CT scans and FMs in the CT image domain, preserving beam geometry for treatment delivery. Instead of splitting CT and FMs into two different input channels, we used single input channel that represents CT and FMs simultaneously to simplify model training process and reduced computational load. In contrast to earlier approaches, this novel integration of CT images with explicitly encoded beam geometry was used as the single input for the 3D U-Net. Compared to existing approaches in literature [44, 47, 48], our method circumvented the requirement for computational-demanding algorithms in data preprocessing, reducing the overall computational time needed. As illustrated in the Result Section, the proposed approach can potentially enable real-time (∼14.7 ms) dose estimation of ultra-high accuracy, as evidence by the high average $\gamma $ passing rate and the small error in dose quantities of interest for both PTVs and OARs. This indicated that our model can adequately learn physics principles between FMs and CT images in dose calculation.

The workflow of online ART often starts with acquiring a volumetric image for the patient on the treatment couch. Based on this image, a synthetic CT (sCT) is often generated so that the treatment plan can be adapted to patient’s anatomy and positioning at the treatment. FMs can then be reconstructed and encoded for the adapted treatment plan following the same FM reconstruction and geometry encoding approach. The FMs and sCT represents the actual treatment beam and patient anatomy in online ART and therefore can be used to estimate the dose and compared with primary dose calculation for the QA purpose.

In addition to its application in online ART dose verification, the proposed DL-based dose estimation algorithm has great potential to accelerate other critical clinical tasks. For instance, it can be employed to boost the efficiency of plan optimization. In treatment plan optimization, the iterative optimization algorithm typically involves a large number of forward dose calculation process, which requires extensive computational efforts and time. By employing the proposed framework, ultra-fast dose estimation can be performed in almost real-time to replace the forward dose calculation process, and thereby reduce the overall computational time of plan optimization to a large extent.

This paper summarizes a proof-of-principle study to investigate the feasibility of the geometry-encoded approach for accurate dose prediction in real time. We acknowledge several limitations of the study in its current form, which can be addressed in future works. The current study primarily focuses on dose estimation for a single 2D slice. Our model’s input, a FM-encoded CT sub-volume, has dimensions of $256 \times 256 \times 16$. However, dose evaluation is restricted to the central axial slice of this sub-volume. We expect that incorporating surrounding slices as part of the input will provide critical dose scattering information, enhancing overall model performance. Theoretically, omitting adjacent slices compromises accuracy, even with the most sophisticated dose calculation engines. While this proof-of-principle study shows potential, relying on single-slice predictions is inadequate for real-world clinical application. We plan to expand our framework to enable comprehensive 3D dose estimation. This expansion will inevitably increase the GPU memory and computational demands. Further research is essential to develop a model capable of delivering accurate, real-time 3D dose estimations without exceeding GPU memory limits during training and inference. Furthermore, the accuracy of predicted doses at the tissue interface, e.g. between air and human tissue, exhibited relatively larger error. This is a common challenge aligning with the findings from other studies published in literature [26, 44, 49]. The inaccurate dose could be attributed to the significant variations in material density present in boundary regions, impacting the performance of the model. Further research investigations down the road are required to mitigate this issue. Additionally, the current training datasets consist exclusively full-arc VMAT plans for prostate cancer. The feasibility of the current model for dose verification for fixed beam intensity-modulated RT (IMRT) needs to be further validated while fine-tuning using IMRT data might be required.

## 5. Conclusion

This study has demonstrated the feasibility of using a unified representation for CT scans and FMs to streamline the DL model training process for RT dose estimation. By integrating CT images with explicitly encoded beam geometry into a single input for the 3D U-Net model, the proposed approach significantly reduces the computational demand associated with data preprocessing. Our results indicate that this novel method can achieve ultra-high accuracy in real-time dose estimation, as evidenced by the high average *γ* passing rate and minimal errors in dose metrics for both PTVs and OARs. The proposed DL-based dose estimation algorithm not only holds promise for online ART dose verification but also has the potential to enhance other critical clinical tasks, such as treatment plan optimization. Future work is needed to extend this framework to 3D dose estimation, which will require increased GPU RAM and computational resources.

## Data Availability

The data cannot be made publicly available upon publication because they contain sensitive personal information. The data that support the findings of this study are available upon reasonable request from the authors.
